# National Prevalence of Disability and Disability Types Among Adults in the US, 2019

**DOI:** 10.1001/jamanetworkopen.2021.30358

**Published:** 2021-10-21

**Authors:** Varshini Varadaraj, Jennifer A. Deal, Jessica Campanile, Nicholas S. Reed, Bonnielin K. Swenor

**Affiliations:** 1 Johns Hopkins Disability Health Research Center, Baltimore, Maryland; 2 Wilmer Eye Institute, Johns Hopkins University School of Medicine, Baltimore, Maryland; 3 Department of Epidemiology, Johns Hopkins Bloomberg School of Public Health, Baltimore, Maryland

## Abstract

This cross-sectional study uses data from the 2019 Behavioral Risk Factor Surveillance System to examine the most recent estimates of disability prevalence among adults in the US.

## Introduction

Updated estimates of disability prevalence, including by race, gender, and other demographic characteristics, are needed to monitor health and to develop public health programs to address the health inequities for the disability community.^1^ Mandated by federal statute, the 6-question sequence on disability (6QS) from the American Community Survey is deemed the minimum data standard to survey disability.^2,3^ To examine the most up-to-date estimates of disability prevalence, we analyzed 2019 Behavioral Risk Factor Surveillance System (BRFSS) data in this cross-sectional study.

## Methods

The BRFSS is a state-based, telephone survey of noninstitutionalized US adults aged 18 years or older.^4^ Verbal informed consent was obtained from participants. The Centers for Disease Control and Prevention determined that the BRFSS protocol is exempt from institutional review board approval. This study followed the Strengthening the Reporting of Observational Studies in Epidemiology (STROBE) reporting guideline for cross-sectional studies.

The 6QS assessed the following disability types: hearing, vision, cognition or mental, mobility, self-care, and independent living (eAppendix in the Supplement). The Sexual Orientation/Gender Identity module was used to examine gender characteristics. Survey-weighted and age-standardized (using the 2010 US census population)^5^ prevalence were calculated for any disability and disability type in Stata statistical software version 14 (StataCorp). Significance was defined at *P* < .05, and 2-sided values are presented. Pearson χ^2^ tests were used to examine demographic differences for any disability. Data analysis was performed from April to May 2021.

Secondary analyses were stratified by 4 race and ethnicity groups (non-Hispanic White, Black, or other [ie, Asian, Native Hawaiian or other Pacific Islander, multiracial, and any other race not specified], and Hispanic). Race and ethnicity were self-reported by study participants using several options defined by the investigator. Race and ethnicity were assessed in this study to examine potential group differences in disability prevalence.

## Results

A total of 418 268 adults (228 433 women [51%]) participated in the 2019 BRFSS. Of them, 26.8% (95% CI, 26.5%-27.0%) of the participants, representing 67.2 million adults, reported any disability, and 11.7% (95% CI, 11.5%-11.9%), representing 29.6 million adults, reported more than 1 disability (Table 1). Mobility was the most prevalent disability type (34.2 million adults; 13.3%; 95% CI, 13.1%-13.5%), followed by cognitive or mental (28.6 million adults; 12.1%; 95% CI, 11.9%-12.3%), independent living (17.6 million adults; 7.2%; 95% CI, 7.0%-7.4%), hearing (16.2 million adults; 6.1%; 95% CI, 6.0%-6.3%), vision (12.8 million adults; 5.2%; 95% CI, 5.0%-5.3%), and self-care (9.8 million adults; 3.9%; 95% CI, 3.8%-4.1%) disabilities. Adults with disability were more likely than those without disability to be older (age ≥75 years, 16.6% [95% CI, 16.3%-17.0%] vs 5.9% [95% CI, 5.8%-6.1%]), female (53.7% [95% CI, 53.0%-54.4%] vs 50.0% [95% CI, 49.7%-50.4%]), and Hispanic (20.8% [95% CI, 20.2%-21.5%] vs 17.2% [95% CI, 16.8%-17.5%]); have less than high school education (20.7% [95% CI, 20.1%-21.3%] vs 9.7% [95% CI, 9.5%-10.0%]) and a lower income (annual household income <$25 000, 44.8% [95% CI, 44.0%-45.5%] vs 19.2% [95% CI, 18.9%-19.6%]); and less likely to be employed (unemployed status, 11.5% [95% CI, 11.0%-12.0%] vs 4.4% [95% CI, 4.2%-4.5%]) (Table 2). People with a disability were also more likely than those without a disability to be bisexual (9.4% [95% CI, 8.7%-10.1%] vs 4.0% [95% CI, 3.8%-4.2%]), transgender (0.8% [95% CI, 0.6%-1.0%] vs 0.3% [95% CI, 0.03%-0.4%]), or gender nonconforming (0.4% [95% CI, 0.3%-0.5%] vs 0.1% [95% CI, 0.1%-0.1%]).

**Table 1.** Disability Estimates in the US Among Adults Aged 18 Years and Older in the Behavioral Risk Factor Surveillance System, 2019^a^

Disability type	Unweighted counts (weighted %) [95% CI] (N = 418 268)	Population size, millions^b^	Age-standardized, weighted % (95% CI)^c^
Disability			
Any^d^	127 138 (27.6) [27.4-27.9]	67.2	26.8 (26.5-27.0)
>1	57 273 (12.1) [12.0-12.3]	29.6	11.7 (11.5-11.9)
Disability type			
Hearing	37 961 (6.7) [6.5-6.8]	16.2	6.1 (6.0-6.3)
Vision	22 403 (5.3) [5.2-5.4]	12.8	5.2 (5.0-5.3)
Cognition or mental	44 665 (11.9) [11.7-12.1]	28.6	12.1 (11.9-12.3)
Mobility	71 550 (14.2) [14.0-14.4]	34.2	13.3 (13.1-13.5)
Self-care	18 574 (4.1) [4.0-4.2]	9.8	3.9 (3.8-4.1)
Independent living	32 099 (7.3) [7.8-7.5]	17.6	7.2 (7.0-7.4)

^a^ Strata with a single sampling unit are treated as certainty units (ie, they do not contribute to SEs).

^b^ Population size in millions are the weighted counts.

^c^ US 2010 Census weights were used for age standardization.

^d^ Participants who responded yes to at least 1 disability question were identified as having any disability.

**Table 2.** Age-Standardized, Weighted, Unadjusted, Disability Prevalence Estimates by Sociodemographic Characteristics and Stratified by Race and Ethnicity, Among Adults Aged 18 Years and Older in the Behavioral Risk Factor Surveillance System, 2019^a^^,^^b^

Characteristics	Weighted % (95% CI)					
	Disability		Any disability by race and ethnicity			
	None (n = 176.1)^c^	Any (n = 67.2)^c^^,^^d^	Hispanic (n = 11.9)^c^	Non-Hispanic Black (n = 8.2)^c^	Non-Hispanic Other (n = 4.6)^c^^,^^e^	Non-Hispanic White (n = 41.0)^c^
Age category, y^f^						
18-29	22.6 (22.3-23.0)	14.9 (14.4-15.3)	21.1 (19.6-22.7)	14.5 (12.9-16.2)	20.7 (18.7-23.0)	12.5 (12.1-13.0)
30-39	20.0 (19.6-20.3)	11.5 (11.1-11.9)	15.6 (14.4-16.9)	12.2 (11.0-13.5)	12.2 (10.8-13.8)	9.9 (9.5-10.4)
40-49	16.4 (16.1-16.6)	11.5 (11.1-11.9)	15.4 (14.1-16.9)	13.4 (12.2-14.6)	13.1 (11.2-15.1)	9.8 (9.4-10.2)
50-64	24.0 (23.7-24.3)	28.6 (28.1-29.1)	26.9 (25.3-28.5)	33.4 (31.9-35.1)	27.3 (25.0-29.8)	28.3 (27.7-28.8)
65-74	11.1 (11.0-11.3)	17.0 (16.7-17.4)	11.9 (10.9-13.0)	15.2 (14.1-16.3)	15.3 (13.5-17.4)	19.1 (18.7-19.5)
≥75	5.9 (5.8-6.1)	16.6 (16.3-17.0)	9.1 (8.1-10.1)	11.3 (10.5-12.3)	11.3 (9.7-13.1)	20.4 (19.9-20.8)
Sex at birth						
Male	50.0 (49.6-50.3)	46.3 (45.6-47.0)	47.0 (45.2-48.8)	41.4 (39.3-43.4)	46.8 (44.1-49.6)	46.4 (45.6-47.2)
Female	50.0 (49.7-50.4)	53.7 (53.0-54.4)	53.0 (51.2-54.9)	58.7 (56.6-60.1)	53.2 (50.4-55.9)	53.6 (52.8-54.4)
Sexual orientation						
Straight	94.2 (93.9-94.5)	88.0 (87.3-88.7)	86.8 (84.6-88.7)	91.7 (90.0-93.0)	86.6 (83.8-89.0)	87.5 (86.6-88.2)
Gay or lesbian	1.8 (1.7-2.0)	2.6 (2.3-2.9)	2.6 (1.9-3.5)	2.5 (1.8-3.4)	2.5 (1.7-3.6)	2.7 (2.4-3.1)
Bisexual or other	4.0 (3.8-4.2)	9.4 (8.7-10.1)	10.7 (8.9-12.8)	5.9 (4.7-7.3)	10.9 (8.7-13.7)	9.8 (9.1-10.6)
Gender identity						
Cisgender	99.6 (99.5-99.7)	98.9 (98.6-99.0)	98.7 (98.0-99.2)	99.5 (99.0-99.7)	98.8 (97.9-99.3)	98.7 (98.4-99.0)
Transgender	0.3 (0.03-0.4)	0.8 (0.6-1.0)	0.8 (0.5-1.5)	0.4 (0.2-0.8)	1.0 (0.5-1.8)	0.9 (0.7-1.2)
Gender nonconforming	0.1 (0.1-0.1)	0.4 (0.3-0.5)	0.4 (0.2-0.9)	0.2 (0.0-0.6)	0.3 (0.1-0.6)	0.4 (0.3-0.6)
Race and ethnicity^g^						
Hispanic	17.2 (16.8-17.5)	20.8 (20.2-21.5)	NA	NA	NA	NA
Non-Hispanic						
Black	11.2 (10.9-11.4)	13.0 (12.5-13.5)				
Other^e^	8.9 (8.7-9.2)	7.5 (7.1-7.9)				
White	62.7 (62.3-63.1)	58.7 (58.0-59.4)				
Education						
Less than high school	9.7 (9.5-10.0)	20.7 (20.1-21.3)	42.7 (41.0-44.4)	22.1 (20.5-23.8)	17.2 (15.1-19.5)	14.3 (13.6-14.9)
High school	26.2 (25.9-26.5)	32.3 (31.6-32.9)	26.3 (24.8-27.9)	36.4 (34.4-38.5)	27.1 (24.9-29.4)	33.6 (32.9-34.4)
Some college	30.9 (30.5-31.2)	31.7 (31.1-32.2)	21.2 (19.7-22.8)	30.2 (28.4-32.1)	32.4 (29.9-35.0)	35.3 (34.5-36.0)
College graduate	33.2 (32.9-33.5)	15.4 (15.0-15.8)	9.9 (9.0-10.8)	11.3 (10.3-12.4)	23.3 (21.2-25.6)	16.8 (16.4-17.3)
Annual household income, $						
<25 000	19.2 (18.9-19.6)	44.8 (44.0-45.5)	60.7 (58.6-62.3)	58.6 (56.4-60.7)	44.1 (41.3-47.0)	36.9 (36.1-37.7)
25 000-50 000	21.7 (21.4-22.1)	24.6 (24.0-25.3)	22.8 (21.1-24.7)	22.9 (21.1-24.9)	23.9 (21.4-26.6)	25.3 (24.5-26.0)
>50 000	59.1 (58.7-59.5)	30.6 (29.9-31.3)	16.5 (15.0-18.1)	18.5 (16.9-20.3)	31.9 (29.2-34.8)	37.9 (37.0-38.7)
Employment status						
Employed for wages	56.6 (56.3-57.0)	45.8 (45.0-46.6)	43.7 (41.7-45.6)	45.4 (43.0-47.8)	42.0 (39.0-45.1)	47.7 (46.8-48.5)
Self-employed	10.8 (10.6-11.1)	9.9 (9.4-10.5)	10.3 (9.1-11.6)	7.8 (6.5-9.4)	10.4 (8.2-13.1)	10.0 (9.4-10.6)
Out of work	4.4 (4.2-4.5)	11.5 (11.0-12.0)	11.1 (9.7-12.5)	15.5 (13.9-17.3)	11.9 (10.1-14.1)	10.8 (10.2-11.4)
Homemaker or student	12.4 (12.1-12.7)	13.1 (12.5-13.7)	18.6 (16.9-20.4)	9.2 (7.5-11.1)	15.0 (12.9-17.4)	11.8 (11.2-12.4)
Retired	15.8 (15.7-16.0)	19.7 (19.4-20.0)	16.4 (15.5-17.4)	22.1 (22.2-23.1)	20.6 (18.9-22.5)	19.8 (19.5-20.2)

Abbreviation: NA, not applicable.

^a^ Strata with a single sampling unit were treated as certainty units (ie, they do not contribute to the SEs).

^b^ US 2010 Census weights were used for age standardization.

^c^ Numbers in parentheses are weighted counts denoting population size in millions.

^d^ Participants who responded yes to at least 1 disability question were identified as having any disability.

^e^ Other refers to Asian, Native Hawaiian or other Pacific Islander, multiracial, and any other race not specified.

^f^ Age estimates shown are survey weighted and not age standardized.

^g^ Race and ethnicity were self-reported by study participants using several options defined by the investigator.

In analyses stratified by race and ethnicity, differences in sociodemographic characteristics were noted between groups. For example, Black women had higher prevalence of disability than women of other races and ethnicities (Black women, 58.7% [95% CI, 56.6%-60.1%]; White women, 53.6% [95% CI, 52.8%-54.4%]; women of other races, 53.2% [95% CI, 50.4%-55.9%]; Hispanic women, 53.0% [95% CI, 51.2%-54.9%]). In addition, compared with Black adults identifying as gay (Black, 2.5% [95% CI, 1.8%-3.4%]; White, 2.7% [95% CI, 2.4%-3.1%]; other, 2.5% [95% CI, 1.7%-3.6%]; Hispanic, 2.6% [95% CI, 1.9%-3.5%]) or bisexual (Black, 5.9% [95% CI, 4.7%-7.3%]; White, 9.8% [95% CI, 9.1%-10.6%]; other races, 10.9% [95% CI, 8.7%-13.7%]; Hispanic, 10.7% [95% CI, 8.9%-12.8%]), gay or bisexual adults of other races and ethnicity had higher prevalence of disability.

## Discussion

More than 1 in 4 noninstitutionalized US adults have a disability, a 1% increase from 2016.^6^ This modest growth in disability prevalence may be due, in part, to aging of the population, as well as increased disclosure of disability status following progress in societal acceptance and legislative protections over time.^1,2^ The Sexual Orientation/Gender Identity module data indicate that bisexual, transgender, and gender nonconforming individuals had a higher prevalence of disability than cisgender individuals. Examination of disability prevalence across racial and ethnic groups further revealed intersectional differences in the age distribution, Sexual Orientation/Gender Identity proportions, and gaps in education, income, and employment.

This study is limited by the fact that these disability estimates are likely conservative and underestimate the true prevalence of disability in the US. For example, BRFSS data are restricted to noninstitutionalized adults, the 6QS ascertains severe impairments, and the telephone-based administration of the survey likely undercounts hearing impairment.

Overall, these findings provide an update on national disability estimates in the US and highlight differences in disability prevalence by race and ethnicity and other demographic variables. As the number of US adults with disabilities increases, the role of surveillance systems like the BRFSS becomes even more critical in identifying health inequities within the disability community. Improved understanding of health inequities for people with disabilities, including across intersecting demographic groups, is needed to develop targeted public health policies and programs that address health inequities for people with disabilities and meet the needs of everyone in our communities.

## Notes

Supplement. **eAppendix.** Six-Question Sequence on Disability (6QS) Questionnaire
