# Supplementary material for: National Prevalence of Disability and Disability Types Among Adults in the US, 2019
Source: JAMA Netw Open. 2021 Oct 21;4(10):e2130358. doi: 10.1001/jamanetworkopen.2021.30358 (PMC8531993; doi:10.1001/jamanetworkopen.2021.30358)
Supplement: Supplement. — eAppendix. Six-Question Sequence on Disability (6QS) Questionnaire [file jamanetwopen-e2130358-s001.pdf]

Supplemental Online Content

Varadaraj V, Deal JA, Campanile J, Reed NS, Swenor BK. National prevalence of disability and disability types among adults in the US, 2019. *JAMA Netw Open*. 2021;4(10):e2130358. doi:10.1001/jamanetworkopen.2021.30358

eAppendix. Six-Question Sequence on Disability (6QS) Questionnaire

This supplemental material has been provided by the authors to give readers additional information about their work.

© 2021 Varadaraj V et al. *JAMA Network Open.*

eAppendix. Six-Question Sequence on Disability (6QS) Questionnaire

1. Are you deaf, or do you have serious difficulty hearing?
2. Are you blind, or do you have serious difficulty seeing, even when wearing glasses?
3. Because of a physical, mental, or emotional condition, do you have serious difficulty concentrating, remembering, or making decisions?
4. Do you have serious difficulty walking or climbing stairs?
5. Do you have difficulty dressing or bathing?
6. Because of a physical, mental, or emotional condition, do you have difficulty doing errands alone such as visiting a doctor’s office or shopping?

Response Options for each question are:
a. ____ Yes
b. ____ No

© 2021 Varadaraj V et al. *JAMA Network Open.*
